# A human population-based organotypic *in vitro* model for cardiotoxicity screening

**DOI:** 10.14573/altex.1805301

**Published:** 2018-07-08

**Authors:** Fabian A. Grimm, Alexander Blanchette, John S. House, Kyle Ferguson, Nan-Hung Hsieh, Chimeddulam Dalaijamts, Alec A. Wright, Blake Anson, Fred A. Wright, Weihsueh A. Chiu, Ivan Rusyn

**Affiliations:** 1 Department of Veterinary Integrative Biosciences, Texas A&M University, College Station, TX, USA;; 2 Bioinformatics Research Center,; 3 Department of Biological Sciences, North Carolina State University, Raleigh, NC, USA;; 4 Department of Statistics, North Carolina State University, Raleigh, NC, USA;; 5 Cellular Dynamics International, Madison, WI, USA

**Keywords:** iPSC, cardiomyocyte, toxicity

## Abstract

Assessing inter-individual variability in responses to xenobiotics remains a substantial challenge, both in drug development with respect to pharmaceuticals and in public health with respect to environmental chemicals. Although approaches exist to characterize pharmacokinetic variability, there are no methods to routinely address pharmacodynamic variability. In this study, we aimed to demonstrate the feasibility of characterizing inter-individual variability in a human *in vitro* model. Specifically, we hypothesized that genetic variability across a population of iPSC-derived cardiomyocytes translates into reproducible variability in both baseline phenotypes and drug responses. We measured baseline and drug-related effects in iPSC-derived cardiomyocytes from 27 healthy donors on kinetic Ca^2+^ flux and high-content live cell imaging. Cells were treated in concentration-response with cardiotoxic drugs: isoproterenol (β-adrenergic receptor agonist/positive inotrope), propranolol (β-adrenergic receptor antagonist/negative inotrope), and cisapride (hERG channel inhibitor/QT prolongation). Cells from four of the 27 donors were further evaluated in terms of baseline and treatment-related gene expression. Reproducibility of phenotypic responses was evaluated across batches and time. iPSC-derived cardiomyocytes exhibited reproducible donor-specific differences in baseline function and drug-induced effects. We demonstrate the feasibility of using a panel of population-based organotypic cells from healthy donors as an animal replacement experimental model. This model can be used to rapidly screen drugs and chemicals for inter-individual variability in cardiotoxicity. This approach demonstrates the feasibility of quantifying inter-individual variability in xenobiotic responses and can be expanded to other cell types for which *in vitro* populations can be derived from iPSCs.

## 1 Introduction

Inter-individual variability in responses to xenobiotics remains a substantial clinical and public health challenge. For pharmaceuticals, inter-individual variability can result in patients either receiving clinically ineffective treatments or suffering harmful side effects (Turner et al., 2015). For environmental toxicants, addressing inter-individual variability is necessary to ensure that exposure limits are protective not only of “typical” individuals, but also of sensitive subpopulations (Zeise et al., 2013). However, population-level data on xenobiotic responses are generally lacking. For instance, clinical trials often fail to identify the most vulnerable individuals and the causes for their enhanced susceptibility, and environmental risk assessments rely predominantly on 10-fold uncertainty factors to ensure inter-individual susceptibility is addressed (Laverty et al., 2011; Meek et al., 2002). Some progress has been achieved in incorporating population pharmacokinetics to address variability in the processes of absorption, distribution, metabolism, and excretion, including use of *in silico* and *in vitro* methods (Jamei, 2016). Indeed, population-based *in vitro-in vivo* extrapolation (IVIVE) to address pharmacokinetic variability is an increasingly common step in safety evaluations for both pharmaceuticals and environmental chemicals (Bell et al., 2017; Wetmore, 2015). However, it is well-recognized that pharmacodynamic processes likely contribute equally, if not more, to inter-individual variability (Turner et al., 2015; Zeise et al., 2013; Hattis and Lynch, 2007). Existing examples of *in vitro* studies aimed at characterizing pharmacodynamic component of population variability for drugs or environmental chemicals have been conducted either in human lymphoblast cell lines (Abdo et al., 2015) or induced pluripotent stem cells (iPSC) derived from the individuals with familial syndromes (Li et al., 2017). The results of these studies demonstrate promise in replicating *in vivo* pharmacodynamic variability (Abdo et al., 2015) and clinical syndromes (Li et al., 2017), but these approaches have not been extensively explored.

iPSC technology has advanced significantly over the past decade and now allows generation of iPSC-derived, differentiated cell types representing a variety of major human tissue types (Kimbrel and Lanza, 2016; Shi et al., 2017; Suh, 2017). As opposed to traditional cell lines, iPSC-derived models retain their organotypicity and provide flexibility for studying both healthy and disease-related phenotypes (Bernstein, 2017; Li et al., 2017; Suter-Dick et al., 2015; Wen, 2017). Second, iPSC-derived cells can be obtained in large quantities which enables their use in screening large numbers of chemicals for many biological readouts (Grimm et al., 2016; Sirenko et al., 2014). iPSCs are a promising *in vitro* alternative to traditional animal testing in pre-clinical efficacy and safety evaluations (Kramer et al., 2016; Shinde et al., 2016; Suter-Dick et al., 2015). Generation of donor-specific cells provides unique opportunities to study genetic variability *in vitro* (Payne et al., 2015; Ronen and Lili, 2016; Smith et al., 2017; Suh, 2017; Wen, 2017).

Conceptually, studies of population genetics are feasible *in vitro* by using iPSCs and iPSC-derived organotypic models (Kilpinen et al., 2017; Panopoulos et al., 2017). While there is evidence that phenotypic responses in undifferentiated iPSCs are directly related to genetic differences between donors, the utility of sensitive physiologic phenotypes for population variability assessment in differentiated iPSC-derived cell types remains largely uncharacterized (Kilpinen et al., 2017; Panopoulos et al., 2017). Therefore, we hypothesize that genetic variability across iPSC-derived differentiated cells will translate into population inter-individual variability in both baseline phenotypes and pharmacodynamic responses to xenobiotic treatment.

We sought to demonstrate this approach using iPSC-derived cardiomyocytes, which have emerged as a particularly attractive and valuable *in vitro* model for cardiotoxicity testing (Blinova et al., 2017; Strauss et al., 2017; Burridge et al., 2016; Sharma et al., 2017; Kawatou et al., 2017). These cells are now used widely for predictive assessment of cardiac arrhythmic potential of drugs and environmental chemicals (Sirenko et al., 2017; Blinova et al., 2017; Pfeiffer et al., 2016); however, no previous study of iPSC-derived cardiomyocytes addressed population inter-individual variability. In this study, the goals were to demonstrate the utility of a human *in vitro* model for the drug and chemical safety endpoint that currently relies heavily on the use of animals (e.g., dogs) and to bridge clinical and experimental paradigms by a model that can be used to address population-based cardiovascular safety. Specifically, we establish (a) that variability in baseline phenotypes and treatment-related responses across individual donors can be attributed to intrinsic biological variability rather than experimental variation; (b) that variability at baseline and with treatment is reproducible; and (c) that the extent of treatment-related variability is chemical- and endpoint-specific. To test these, we conducted high throughput/high content screening of commercially available iPSC-derived cardiomyocytes from 27 donors with no known cardiovascular disease. Our screening approach included media- and vehicle controls as well as treatments at four concentrations with three drugs of known cardiotoxic mechanisms: the β-adrenergic receptor agonist isoproterenol (ISO, positive inotrope), the β-adrenergic receptor antagonist propranolol (PRO, negative inotrope), and the hERG channel inhibitor cisapride (CIS, QT prolongation). Readouts included high-content imaging of Ca^2+^ flux and markers of cytotoxicity and mitochondria integrity, in addition to targeted high-throughput transcriptomics for 4 donors.

## 2 Materials and Methods

### 2.1 Chemicals and biologicals

Cardiomyocyte plating and maintenance media were obtained from Cellular Dynamics International (CDI, Madison, WI). Tissue culture grade dimethyl sulfoxide (DMSO, CAS: 67–68-5) was from Santa Cruz Biotechnology (Dallas, TX). Trypan Blue (0.4%) and penicillin/streptomycin (50 mg/ml) were from Life Technologies (Grand Island, NY). Isoproterenol (CAS: 7683–59-2) and propranolol (CAS: 525–66-6) were from Molecular Devices (Sunnyvale, CA); cisapride (CAS: 81098–60-4) was from Sigma Aldrich (St. Louis, MO). Phosphate buffer saline (PBS), LC/MS grade acetonitrile, LC/MS grade water with 0.1% formic acid were from Fisher Scientific (Waltham, MA). Human plasma was from Bioreclamation (Westbury, NY) and all donors of plasma tested negative for viral antigens.

### 2.2 iPSC-derived cardiomyocytes

iCell cardiomyocytes (cat. no: CMC-100–010-001), and MyCell cardiomyocyte products were from CDI. Cells were engineered from donor plasma samples obtained from CDI’s own repository (iCell products) or the National Heart, Lung, and Blood Institute (MyCell products). The donor population consisted of 12 females and 15 males, of which 85% were of Caucasian (n=23) and 15% African American (n=4) ancestry. A summary of donor IDs and demographic information can be found in Table 1.

### 2.3 Cell culture

iCell and MyCell cardiomyocytes were cultured under identical conditions in multiple batches using an established protocol (Grimm et al., 2015). Cardiomyocytes were thawed for 4 min at 37 °C, resuspended in 30 ml cardiomyocyte plating medium containing penicillin/streptomycin. Live cell counts were confirmed using trypan blue exclusion and cells were further diluted to a final concentration of 2×10^5^ cells/ml. Tissue-culture treated microplates (cat.no.: 353962, Corning Life Sciences, Corning, NY) were gelatinized for 2 hours at 37°C with 25μl 0.1% gelatin in water. Cell suspension (25 μl) was dispensed into each well of a 384-well microplate (5000 cells/well). Cells were incubated at 37 °C and 5% CO_2_ for 48 hours. The plating medium was then exchanged with 40 μl of maintenance medium containing penicillin/streptomycin per well. Maintenance medium was replaced every 48–72 hours until day 13 post-plating. The maintenance medium was then exchanged with 50 μl fresh medium per well. Cells were treated the next morning (day 14 post-plating) with 12.5 μl 5x chemical solutions in 0.5% (v/v) of DMSO in media (vehicle) in addition to untreated or vehicle-treated negative control wells, and incubated at 37°C and 5% CO_2_. Following 24 hours of incubation, the cell medium was discarded, and cardiomyocytes were lysed with 10μl 1x lysis buffer provided in the TempO-Seq assay kit (BioSpyder Technologies, Carlsbad, CA). Lysate-containing micro-plates were stored at −80°C.

### 2.4 Protein binding

To confirm and compare unbound drug concentrations, plasma protein and medium protein binding were evaluated for each drug utilizing rapid equilibrium dialysis (RED) as described elsewhere (Wetmore et al., 2012; Waters et al., 2008). The RED assay was conducted using single use RED inserts (cat. no. 90006, Pierce Biotechnology, Rockford, IL) according to instructions, with protocol modification to incorporate “no protein” equilibrium controls. Equilibrium controls comprising of PBS buffer in both sample and buffer chambers were used to ensure drugs are fully equilibrated within the device in the absence of proteins. All RED assays were completed in triplicate. Drug concentrations were measured using an Agilent (Santa Clara, CA) 6470 triple quadrupole mass spectrometer operating in positive ion mode with a Waters Acquity H class HPLC (Milford, MA). Samples were spiked with a known amount of internal standard sotalol (CAS: 959–24-0) prior to analysis. Chromatographic separation was achieved using a linear acetonitrile gradient on a C18 column (Agilent Zorbex Eclipse Plus C18 3.0 X 50 mm, 1.8 μm) with a C18 guard column (Agilent). The mobile phase consisted of 0.1% formic acid, the flow rate was kept at a constant 0.4 μl/min. Complete mass spec conditions for the three drugs are listed in Table S1^1^.

### 2.5 Ca^2+^ flux assay

The Ca^2+^ flux assay was used to evaluate functional performance of cardiomyocytes (Grimm et al., 2015; Grimm et al., 2016; Sirenko et al., 2013a, 2013b, 2017). The assay was initiated by addition of 25 μl pre-warmed Ca^2+^ dye reagent to cardiomyocytes in a total volume of 25 μl maintenance medium. Following 2 hrs of equilibration at 37°C and 5% CO_2_, all sample wells were simultaneously exposed to 12.5 μl of 5x concentrated test chemicals in 2.5% (v/v) DMSO in maintenance medium using the FLIPR^®^tetra cellular screening system with internal liquid handler (Molecular Devices). Exposed cells were kept in the incubator (37°C and 5% CO2) and the intracellular Ca^2+^ flux was recorded 15, 30, 60, and 90 min following initial exposure in the FLIPR^®^tetra, conditioned at 37°C for 100 sec intervals at a sampling frequency of 8 Hz (λ_exc_=470–495 nm, λ_em_=515–575 nm).

### 2.6 Peak processing

Ca^2+^ flux data were analyzed in R studio (version 1.0.136, with R version 3.3.2) to estimate relevant beating parameters. Specifically, for each 100 sec of Ca^2+^ flux data, the mean and CV of the peak height (distance from baseline to peak), mean of peak frequency (beats per minute), CV of peak spacing (time between peaks), and mean ratio of the decay time (time from peak to baseline) to the rise time (time from baseline to peak) were estimated. Decreases in peak height are indicative of either cytotoxicity or inhibition of beating (e.g., quiescence or Torsade’s when accompanied by increased peak frequency); changes in peak frequency (in the absence of decreases in peak height) are indicative of positive or negative inotropes; and increases in the CV of either peak height or peak spacing are indicative of irregular beating. QT prolongation is indicated by an increase in the decay/rise ratio, as this reflects a delay in the ability to repolarize the action potential and start another beat. The use of the ratio adjusts for the fact that slower beating alone increases the decay time, but not the decay/rise ratio. Additionally, the analysis notes when a “notch” is present in which the Ca^2+^ flux partially declines, then “plateaus” for a period before completely returning to baseline. Further analysis was conducted with beating parameters evaluated at 90 minutes after treatment along with high-content cell imaging data (below). Beating parameters at earlier time points were examined for comparison.

### 2.7 High-content cell imaging

Cytotoxicity and mitochondrial integrity were quantitatively assessed by high-content imaging after conclusion of Ca^2+^ flux measurements at 90 min and prior to TempO-seq lysate preparation at 24 hrs as described previously (Grimm et al., 2016). Briefly, cell culture medium including (90 min) or excluding (24 hrs) Ca^2+^ dye was replaced with 25 μl of staining solution (2 μg/ml Hoechst 33342 and 0.2 μM MitoTracker Orange in iCell cardiomyocyte maintenance medium). Following 15 min incubation at 37°C and 5% CO_2_ the staining solution was discarded and replaced with an equal volume of fresh maintenance medium. Images were then acquired using an ImageXpress Micro Confocal cellular imaging system (Molecular Devices), using DAPI and Cy3 filters for Hoechst 33342 and MitoTracker Orange, respectively. Images were processed using the instrument-specific MetaXpress software package (Molecular Devices). Quantification of imaging-based parameters for concentration-response assessment was achieved using the multi-wavelength cell scoring application module.

### 2.8 Inter-individual variability at baseline and with treatment

Each of the five Ca^2+^ flux-based beating parameters, along with the imaging-based measurement of total cells, were analyzed to determine the contribution and reproducibility of inter-individual variability. For baseline variability, data from vehicle- and media-only controls were analyzed using a linear mixed effects model to evaluate the contribution of biological (i.e., donor) and technical (i.e., inter-plate, and media vs. vehicle) variability to overall variability. For treatment-related variability, concentration-response modeling was performed using a non-linear mixed effects log-logistic model, and only for the “prototypical” endpoint (peak frequency for PRO and ISO, decay-rise ratio for CIS). Treatment-related effects were first normalized to the median control values to control for baseline variability, so all models had control values fixed at 1. For PRO, the expected effect is a decline in beat rate, so a two-parameter model was used in which the high concentration asymptote is fixed at 0: y=(1−(1+exp((ln EC_50_−ln x)/scale)))+*err*, where x is concentration and *err* is the residual error. The EC_50_ was assumed to have both fixed and random effects, whereas the scale (inverse of the Hill coefficient) was assumed only to have a fixed effect. For ISO and CIS, the lowest tested concentration of 0.1 μM already reached a maximal response, so the EC_50_ was set to the nominally lower value 0.001 μM and the scale fixed at 1. The resulting model for ISO was: y=1+(B−1)/(1+exp(lnEC_50_−lnx))+*err*. For CIS, the decay-rise ratio was first natural-log transformed, so the model was: ln y=(B−1)/(1+exp(lnEC_50_−ln x))+*err*. In both cases, the maximal effect B was assumed to have both fixed and random effects. In each case, inter-individual variability in sensitivity was characterized by estimating the relative change from baseline at a nominal concentration of 1 μM.

To assess the reproducibility of inherent cardiomyocyte characteristics, four donors (ID#s 1083, 1134, 1308, and 1434) were selected to represent a wide range of baseline beat rates. New batches of iPSC cardiomyocytes were obtained for these donors and cells were screened approximately 1 year after the initial experiments. Year-to-year reproducibility was evaluated in both baseline and treatment-related effects.

### 2.9 TempO-Seq Gene Expression Profiling

Gene expression was analyzed using TempO-Seq (BioSpyder Technologies) with a targeted RNA sequencing panel comprising 2982 transcripts (House et al., 2017). The sequencing libraries were prepared according to the manufacturer’s instructions. The TempO-Seq libraries were processed using a PCR clean-up kit (Clontech, Mountain View, CA) prior to sequencing using a 50 single-end read mode in a rapid flow cell (2 lanes) on a HiSeq 2500 sequencer (Illumina, San Diego, CA). Sequencing readouts were demultiplexed to generate FASTQ files, and passed all internal quality controls (House et al., 2017). Demultiplexed FASTQ files were processed to generate an expression count matrix using the *temposeqcount* application (House et al., 2017). Count normalization, differential gene expression was conducted using DESeq2 (Love et al., 2014). For comparisons of gene expression to human tissue-specific gene expression from GTEx (GTEx Consortium, 2015), normalized counts for each cardiomyocyte donor were combined with median tissue counts downloaded from the GTEx portal and overlapping genes were kept (n=2640). The overlapping count matrix was scaled by features and samples, and a Pearson correlation matrix was calculated and for average linkage clustering (Galili, 2015). Pathway analysis of differential gene expression patterns for mechanistic interpretation were conducted using Reactome (Fabregat et al., 2016).

## 3 Results

### 3.1 Reference chemicals are present in comparable free concentrations in cardiomyocyte media to those in human plasma

All three test pharmaceuticals fully equilibrated within the RED device in the absence of proteins. Using human serum, CIS was found to be the most highly bound drug (3.9%±0.7% free), as compared to PRO (33.5%±7.6% free) and ISO (56.4%±13.2% free). These measurements are somewhat larger than (limited) reported free fractions in the literature (2–3% for CIS, 10% for PRO, and 35% for ISO), but exhibit the same trend (Kelly and McDevitt, 1978). The unbound drug concentrations in iCell cardiomyocyte maintenance medium were higher still for CIS and PRO (CIS: 57.3%±3.9%; PRO: 78.5%±16.8%), but were lower for ISO (16.3%±4.2%).

### 3.2 iPSC-derived cardiomyocytes exhibit reproducible inter-individual variability in baseline phenotypic characteristics

**Fig. 1:**
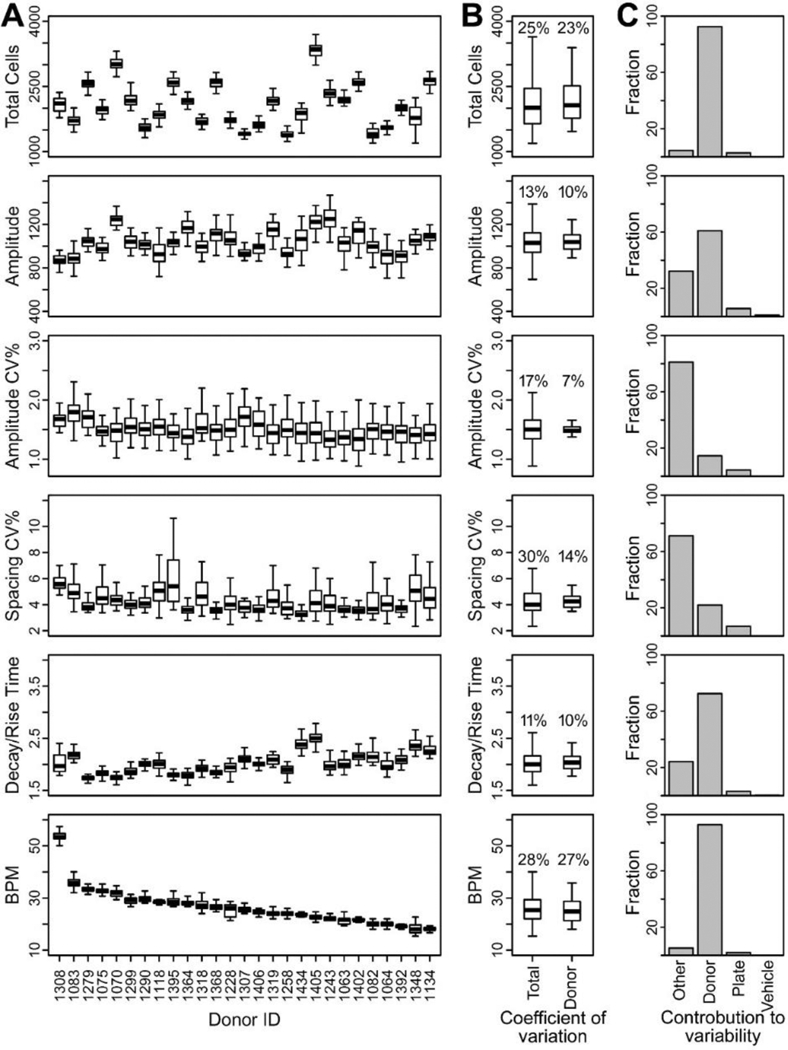
Inter-individual variability in baseline beating parameters of iPSC cardiomyocytes from 27 human donors (A) Boxplots of the median, interquartile range (IQR), and 95% confidence interval (CI) for each donor. (B) Distribution of the coefficient of variation (median, IQR, and min/max) for total (i.e. technical and biological) and biological (“donor”) contributions. (C) Histograms of relative technical and biological contributions to total observable variability for each phenotype [Other=intra-plate variability; Donor=diversity between donors; Plate=inter-plate variability; Vehicle=difference in the effects of 0.5% DMSO vehicle and cell culture media).

iPSC-derived cardiomyocytes exhibited a range of donor-specific differences in their beating characteristics (Figure 1A). For instance, average peak frequency varied reproducibly from <20 beats per minute (BPM) to >50 BPM. Contributions from different sources of variability are shown in Figures 1B–C. Inter-individual variability was the dominant contributor to overall variability for the peak frequency (27% CV donor vs. 28% CV total), decay/rise time (10% vs. 11%) and peak amplitude (10% vs. 13%), with very little contribution from technical variability (plate, vehicle, residuals).

At baseline, cardiomyocyte beating is very regular, peak irregularity measures were generally small (<6% CV for peak spacing and <2% CV for peak amplitude in most cases), with overall variability largely attributable to residual variability, with relatively small contributions from variation across donors. The decay/rise time was not correlated with the beat rate (Fig S1^1^), supporting its use as a surrogate QTc, which adjusts for differences in heart rate. However, the relative plating density (shown as “total cells” in Figure 1), a parameter that is reflective of the plating efficiency between batches of cells, was not completely independent of the Amplitude at baseline (correlation coefficient of 0.55).

**Fig. 2:**
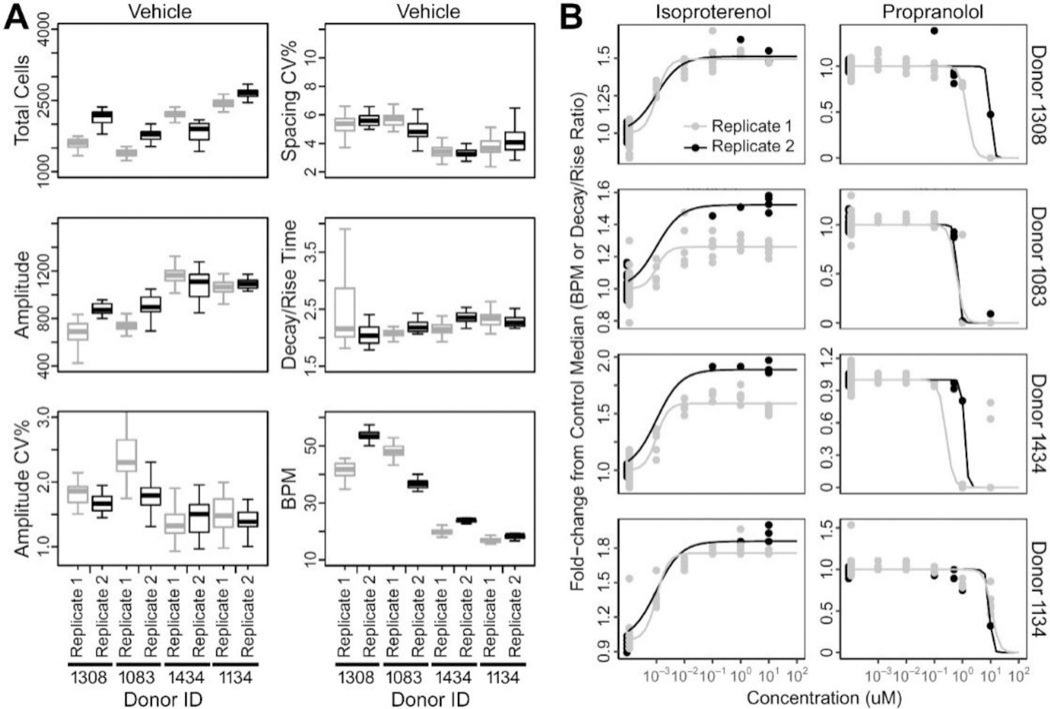
Reproducibility in the baseline (A) and drug- induced (B) beating parameters of iPSC-derived cardiomyocytes derived from different normal donors Replicate experiments were performed at least 6 months apart on four cell lines selected to represent a range of beating frequency among the donors tested (see Figure 1A). **(A)** Box and whisker plots (median, IQR, and min/max) show the range of intra-donor variability. **(B)** The effects of isoproterenol and propranolol in concentration-response are shown across same four donors. Dots are results of the individual wells and lines are a logistic fit to the data.

In addition, when baseline beating parameters were compared across the two batches, experiments performed 6–12 months apart, and demonstrated good reproducibility (Figure 2A). For instance, cells derived from donors 1134 and 1434 consistently beat slower than cells from donors 1083 and 1308. Trends were identical for both media-only and vehicle-exposed cells.

### 3.3 The degree of inter-individual variability in responses to treatment is reproducible, and depends on chemical and phenotypic endpoint

**Fig. 3:**
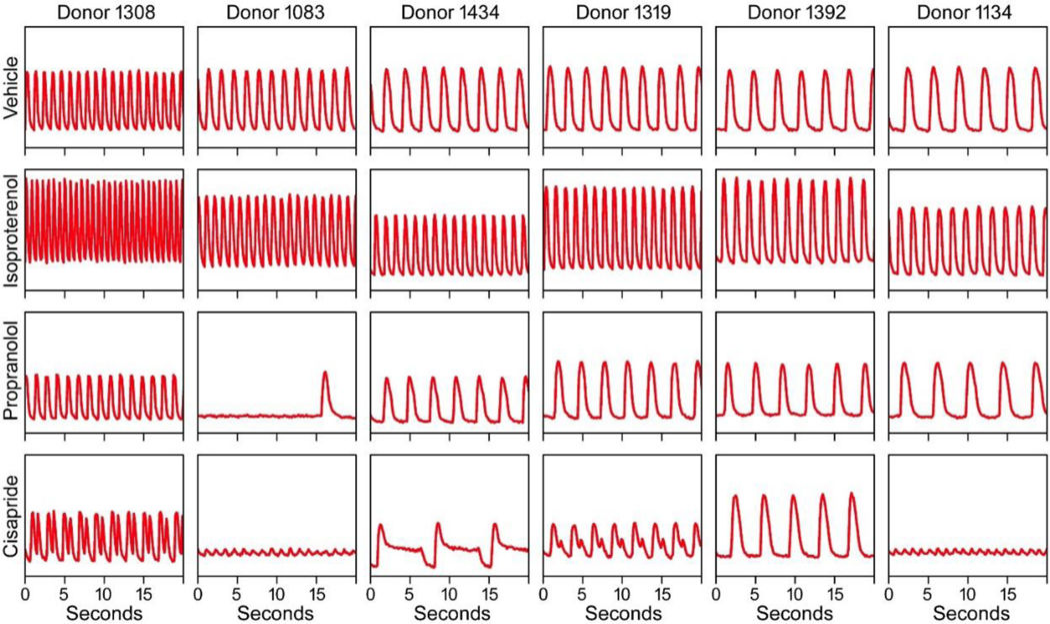
Representative Ca^2+^ flux traces for cells derived from four individual donors Six donors are shown as columns. Rows are organized according to treatments, i.e. baseline (“vehicle”), positive inotrope (“Isoproterenol”), negative inotrope (“Propranolol”), and hERG channel inhibitor (“Cisapride”). Y-axis is showing relative fluorescence units of Ca^2+^ flux that were scaled within each treatment.

**Fig. 4:**
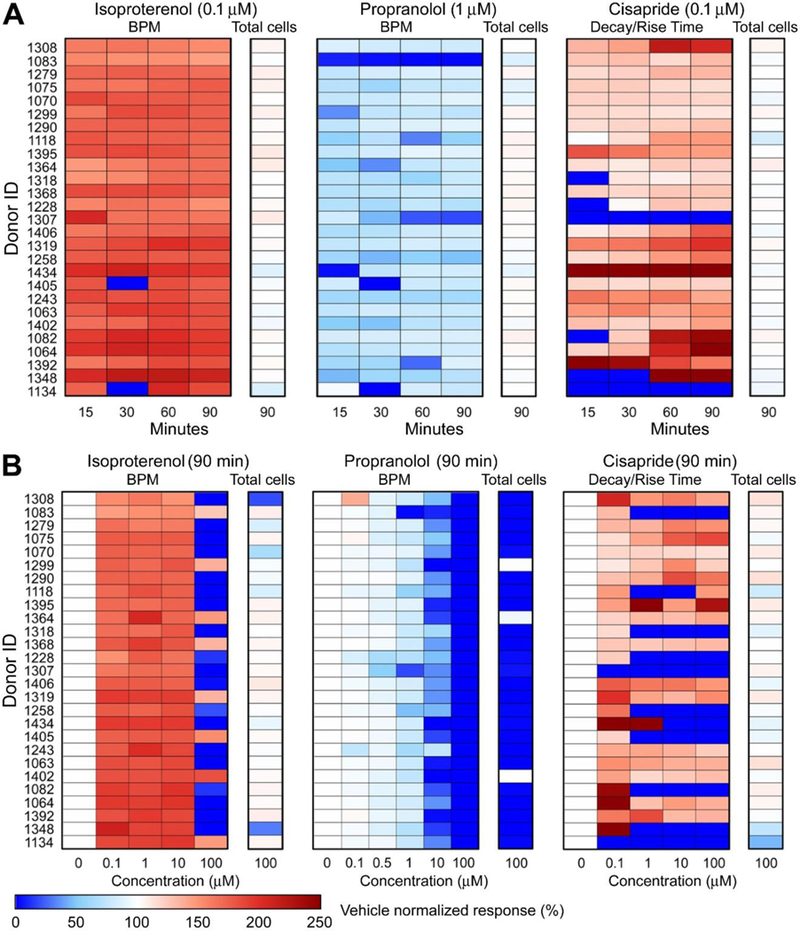
Heatmaps of treatment-related effects at different time-points and concentrations (A) Time-dependent response measurements post-treatment by concentrations shown in Figure 3 (0.1 μM for ISO, CIS; 1 μM for PRO). Results shown are for each drug’s representative cardiophysiologic phenotypes: ISO (BPM), PRO (BPM), and CIS (Decay/Rise Time Ratio). Data are normalized to vehicle controls (0.5% DMSO in media) for each individual donor. Total cell counts are also indicated. (B) The corresponding concentration-response relationships at 90 minutes post-treatment.

The effects of chemical treatments were assessed for the phenotypes associated representative of the pharmacological effect of each compound: increases and decreases in beat frequency for ISO and PRO, and increased decay/rise time ratio (indicative of QT prolongation) for CIS. In each case, cardiomyocyte beating and cell viability were simultaneously monitored. Figure 3 highlights differences in Ca^2+^ flux traces for six donors representing a range of baseline peak frequencies. These six donors include the four selected for year-to-year replication and the four selected for transcriptomic analysis (two overlapped). All donors responded as expected to ISO and PRO, exhibiting increased and decreased BPM, respectively. The effect of PRO was markedly greater in donor 1083, with only a single beat observed. In contrast, treatment with 0.1 μM cisapride revealed a much wider range of differences for the selected donors, including regular QT-prolongation (donor 1434), QT-prolongation with a secondary depolarization peak (donors 1308 and 1319), rapid beating with significant decreases in peak amplitude indicative of Torsade’s (donors 1083 and 1134), and increases in the decay/rise time ratio without characteristic QT “notch” formation (donor 1392). Measurement at 90 min after treatment were generally consistent with those at earlier time points (Figure 4A). In some cases, there appeared to be more variability at earlier time points, with some cell lines undergoing a transient quiescence at 15 or 30 min before “recovering.”

**Fig. 5:**
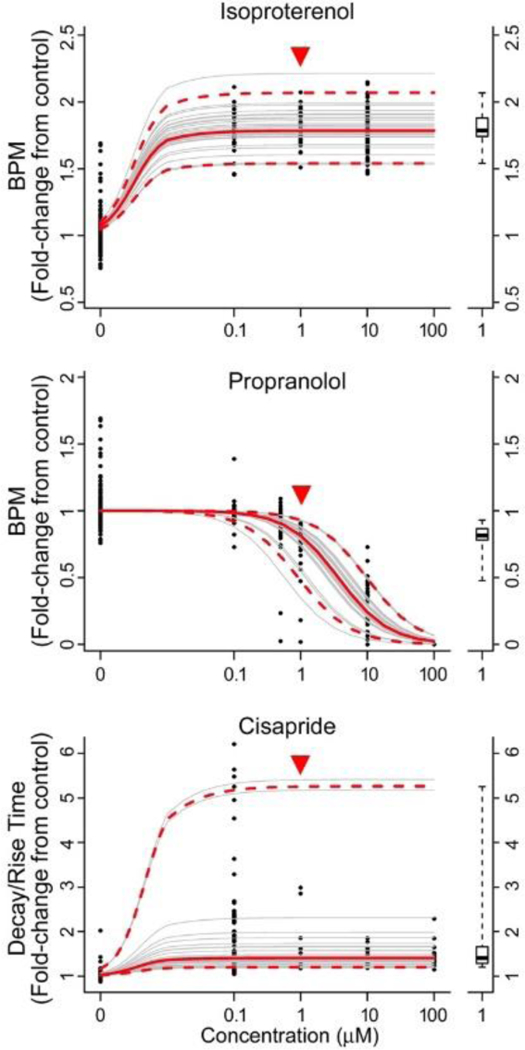
Concentration-response modeling For each representative drug and corresponding reference phenotype, each panel shows the concentration-response data (dots) and non-linear mixed effect model fits for each donor (thin grey lines) and their median (thick red solid lines) and 95% confidence interval (thick red dashed lines). All data are normalized to the median of the vehicle controls for the corresponding donor. The distribution of response predictions at 1 μM (demarked by the inverted red triangle) is shown separately to the right as a box plot (median, interquartile range, and 95% confidence interval).

Next, we evaluated the concentration-response relationships for ISO, PRO, and CIS treatment across all 27 individuals. Heatmaps (Figure 4B) show concentration-response along with cell viability data at the highest tested concentration. For ISO and PRO, many cells became quiescent at 100 μM, with donors (particularly for PRO) showing loss of viability as measured by total cell counts. For CIS, although many donors became quiescent at concentrations as low as 0.1 or 1 μM, total cell counts indicated that cells were still viable. Therefore, for ISO and PRO, the 100 μM group was dropped from the concentration-response modeling (Figure 5), whereas all concentration groups were fit for CIS. As shown in Figure 5, inter-individual variability with treatment was smaller for ISO and PRO, for which change in BPM between vehicle and 1 μM treatment ranged from +50% to +120% and from −7% to −65%, respectively. Inter-individual variability was greater for CIS, which exhibited changes in decay/rise ratio with 1 μM treatment ranging from +19% to +440%. Donor-specific concentration-response relationships were reproducible across year-to-year replicates (Figure 2B).

### 3.4 Gene expression profiling of donor variability at baseline and upon treatment with drugs

**Fig. 6:**
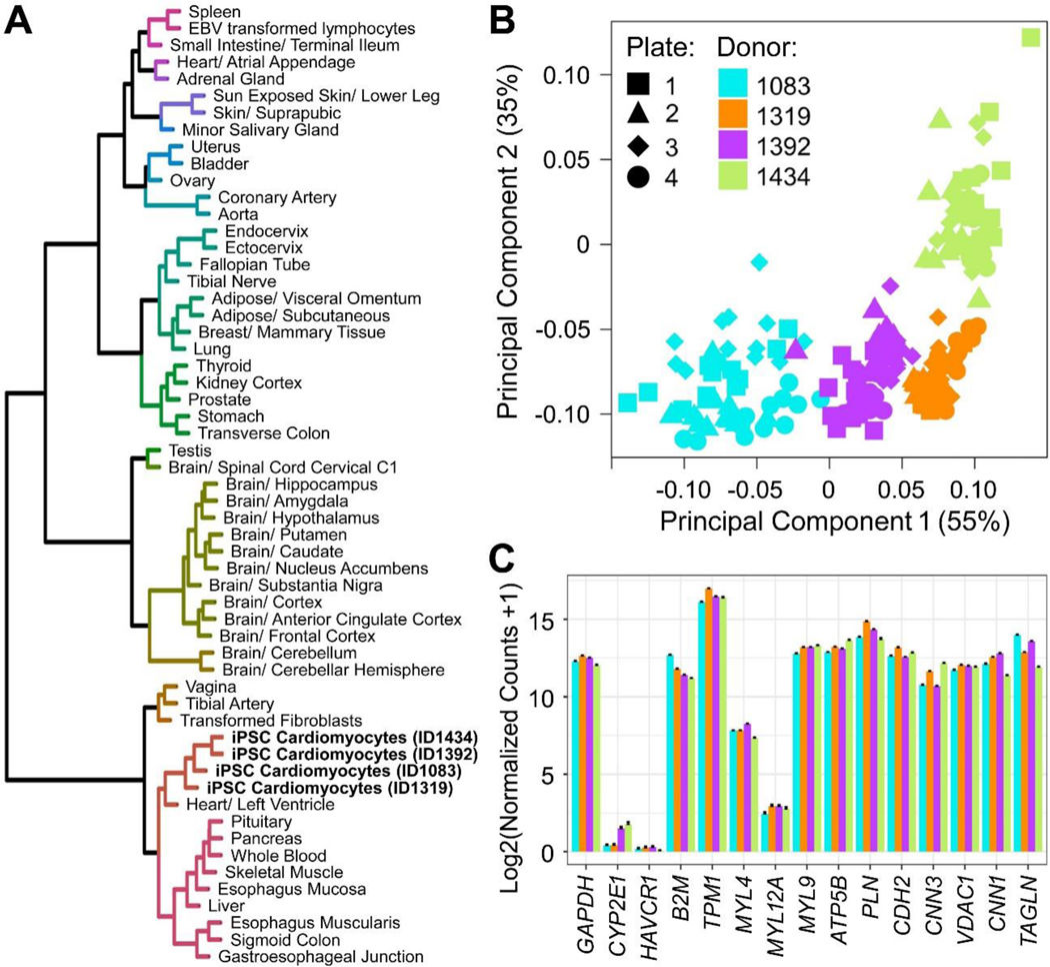
Baseline transcriptomic characterization of iPSC cardiomyocytes from 4 donors **(A)** Expression clustering of donor-specific vehicle treated iPSC cardiomyocytes with each other and with heart tissue from overlapping genes in human tissues from GTEx Consortium (GTEx Consortium et al., 2017). **(B)** Principal components analysis of normalized counts (vehicle controls) from 4 donors of iPSC cardiomyocytes. **(C)** Baseline gene expression levels of cardiac-centric genes in 4 donors of iPSC cardiomyocytes with the *GAPDH* housekeeping gene and two negative controls for heart tissue *(CYP2E1, HAVCR1)*.

For assessment of inter-individual variability in gene-expression, we evaluated expression of a targeted gene set in iPSC-derived cardiomyocytes from four donors (1083, 1319, 1392 and 1434) that are represented in Figure 3. At baseline, vehicle controls for all 4 donors, when compared to median expression of overlapping genes from the GTEx dataset of numerous human tissues,(GTEx Consortium, 2015) clustered together with heart/ventricle tissue (Figure 6A). However, baseline gene expression profiles of these 4 donors were clearly distinguishable from each other (Figure 6B). Illustrating the organotypic nature of these iPSC-derived cells, cardiomyocyte-specific genes were among the most highly expressed in these samples (Figure 6C), whereas expression of genes not expressed in the heart, e.g. *CYP2E1* and *HAVCR1*, was negligible.

**Fig. 7:**
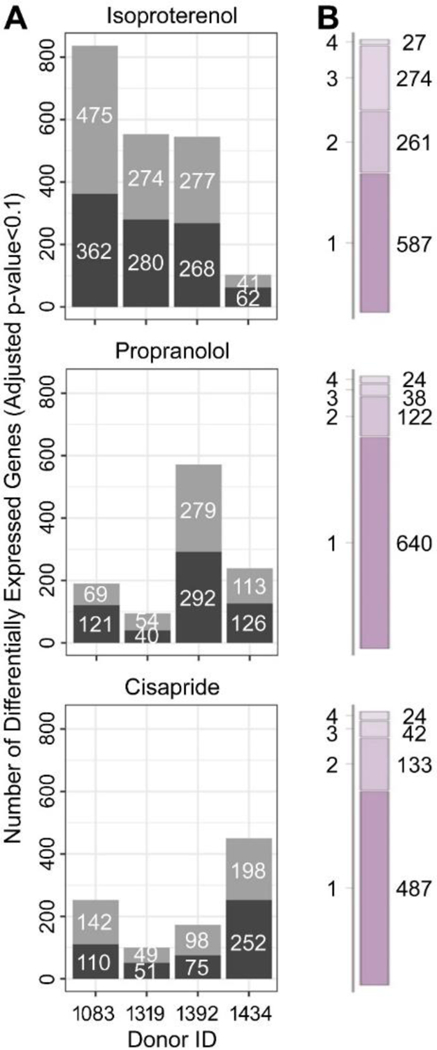
Drug-induced gene expression differences between donors **(A)** The number of up-regulated (grey) and down-regulated (black) genes (false discovery *q*-value < 0.10) when comparing 10 μM drug (ISO, PRO, CIS) to vehicle controls for iPSC cardiomyocytes from donors 1083, 1319, 1392 and 1434. **(B)** The number of differentially expressed genes specific to a single donor, or shared by 2, 3 or4 donors in response to ISO (top) PRO (middle) and CIS (bottom). Corresponding Venn diagrams of overlapping differentially expressed gene sets by iPSC cardiomyocyte donor are included as Figure S2^1^.

Next, we examined inter-individual variability in drug-induced (10 μM vs. vehicle) differential gene expression. The individual donors, as shown in the apical endpoint responses, also exhibited considerable inter-individual differences in their transcriptomic responses to the 10 μM concentrations of isoproterenol, propranolol and cisapride (Figure 7). Donor 1083 exhibited the greatest number of affected transcripts in response to isoproterenol, while donor 1392 was most sensitive to propranolol, and donor 1434 to cisapride (Figure 7A). For each treatment the overlapping gene sets were largely unique (Figure 7B, Fig. S2^1^).

There were 18 genes related to cardiac conduction (based on the classifications in the Reactome database) among the ~3,000 transcripts interrogated by the TempO-seq assay. Of these, 7 were found to be differentially affected among the donors and treatments. For individual 1083, calmodulin 3 (*CALM3*) and phospholamban (*PLN*) were differentially expressed following treatment with isoproterenol. In individual 1319, differential expression of cardiac conduction related genes inositol 1,4,5-trisphosphate receptor, type 2 *(ITPR2)* and ATPase Na+/K+ transporting subunit beta 1 *(ATP1B1)* were observed following cisapride treatment, and potassium channel subfamily K member 1 *(KCNK1)* with isoproterenol treatment. *KCNK1* was differentially expressed following treatment with cisapride in individual 1392. In addition, treatment with propranolol in this individual resulted in differential expression of *ITPR2, CALM3, KCNK1*, and ATPase Na+/K+ transporting subunit alpha 1 *(ATP1A1)*. Calcium/calmodulin-dependent protein kinase type II beta chain *(CAMK2B)* was differentially expressed in cisapride-, isoproterenol, and propranolol-treated cardiomyocytes from individual 1434. The catalytic subunit α of protein kinase A *(PRKACA)* was also differentially expressed following exposure to isoproterenol in this individual.

## 4 Discussion

In this study, we demonstrate the feasibility of using iPSC-derived cardiomyocytes as a human population-based *in vitro* model for inter-individual variability in both baseline and drug-induced cardiac physiologic phenotypes. Using iPSC-derived cardiomyocytes from 27 healthy donors we characterize both baseline variability in cardiophysiologic phenotypes and inter-individual differences in responses to treatment with three reference drugs. While most studies to date have been largely limited to addressing population pharmacokinetics, our results suggest iPSC-derived cardiomyocytes derived from different donors can address the critical need for characterizing inter-individual variability in responses to chemicals (i.e., pharmacodynamics).

Our first key finding was that untreated cardiomyocytes, when cultured using highly standardized procedures and reagents, exhibit both highly reproducible intra-individual phenotypes as well as highly-concordant inter-individual variability in phenotypic characteristics. Specifically, cardiomyocyte beat frequency, decay/rise ratio (a surrogate for QT prolongation), and other characteristics vary considerably in untreated cells across the 27 tested individuals in a reproducible manner, indicating cardiophysiologic performance is a donor-specific biological trait. These data also indicate that cell lines are relatively homogeneous in their lack of baseline arrhythmia, as expected due to these cells being derived from “normal” individuals without known congenital cardiovascular disease. Additionally, inter-individual differences were shown to be consistent between two separate experiments conducted in different years using different batches of cells. Finally, it should be noted that there was no observable correlation between cardiophysiologic responses and either sex or origin/ancestry of the donors (data not shown). Data on the donor age were not available for analysis.

Our second key finding was that the degree of inter-individual variability in responses to treatment depends reproducibly on chemical and phenotypic endpoints. In cardiomyocytes derived from the standard commercial iCell cardiomyocyte product offered by Cellular Dynamics International (individual 1434), the results for our selected reference compounds are in excellent agreement with previous reports (Grimm et al., 2015; Sirenko et al., 2013a,b, 2017). Population-level differences in drug response are evident and the degree of inter-individual variability in responses differ depending on chemical and endpoint. For instance, ISO and PRO cause similar proportional increases/decreases in the beat rate across individuals, while CIS exhibits both qualitative and quantitative phenotypic variation across individuals. Moreover, as with baseline phenotypes, we replicated these inter-individual differences in treatment-related responses in two separate experiments conducted in different years using different batches of cells. These observations strongly suggest that inter-individual variability is a drug-specific characteristic, demonstrating a critical needto characterize inter-individual variability in pharmacodynamic responses in a chemical- and endpoint-specific manner. Moreover, our results demonstrate that population-based *in vitro* models can provide reproducible data to address this need (Meek et al., 2002; Chiu and Rusyn, 2018).

Our third key finding was that the phenotypic observations were corroborated by gene expression changes. Specifically, high-throughput transcriptomics characterized inter-individual variation in gene expression signatures of three drugs in four representative donors. Functional gene expression and similarity assessment revealed biological resemblance to heart tissue, especially of the ventricular type, a finding that is consistent with previous studies and that confirms the organotypicity of these cells (Ma et al., 2011). Despite overall similar expression profiles for cardiac-specific genes, untreated control samples from different individuals were markedly easy to distinguish from each other using normalized gene counts for all genes, indicating distinct differences in baseline gene expression. Gene expression changes after chemical treatments also reflect donor-specific responses. Pathway enrichment analysis in Reactome revealed that many genes involved in cardiac effects were differentially expressed in various donor/treatment combinations and thus demonstrates the potential utility of this platform for mechanistic profiling in a high-throughput format.

This study has notable limitations. First, we were limited to the availability of iPSC-derived cardiomyocytes from 27 individuals, which limits inferences as to the frequency of more extreme sensitivities. Nonetheless, based on recent work employing a Bayesian methodology, this number of individuals appears to be enough to distinguish between “high” and “low” variability cases, and can therefore be used as an initial “screen” to identify compounds with high potential for inter-individual variability (Chiu et al., 2017). Future testing could include larger populations, as several populations of iPSCs with more than 200 individuals each have been reported in the literature (Panopoulos et al., 2017; Kilpinen et al., 2017). However, none have yet reported producing cardiomyocytes across their entire iPSC population. A second limitation is that our demonstration employed only three representative reference compounds. We are currently examining the generalizability of our findings by analyzing the results of testing the same 27 individuals using a larger collection of ~140 compounds, including both pharmaceuticals and environmental contaminants. Third, we have limited our testing to cells derived from “apparently healthy” individuals, whereas in some cases co-morbidities may contribute substantially to population inter-individual variability (Zeise et al., 2013). However, there is no *a priori* reason why similar tests could not be performed in cells derived from individuals with congenital disorders. In the meantime, the degree of inter-individual variability assessed using only “apparently healthy” individuals can be viewed as a lower bound on the overall inter-individual variability in the general population, which includes “unhealthy” individuals with possibly more extreme responses. Finally, our work is limited to one differentiated cell type – cardiomyocytes. Extending to other cell types needs to await the routine availability of consistently differentiated cells from multiple iPSC donors.

In conclusion, we have demonstrated that a human population-based *in vitro* cardiotoxicity model can be used to characterize inter-individual responses in untreated and chemical-treated iPSC-derived cardiomyocytes, an approach that is both useful and feasible. Specifically, high-content screening data indicate that cardiophysiologic phenotypes are reproducible, intrinsic characteristics of iPSC-derived cardiomyocytes, thus confirming that donor-specific characteristics are reflected in the donor-associated phenotype. Moreover, population-variability assessment of chemical exposure in concentration-response for three reference drugs with known mechanisms of action and cardiophysiological effects revealed chemical-specific variation in population-level responses, demonstrating the need for chemical-specific data on inter-individual variability. For pharmaceuticals, this issue will likely be of particular concern for drugs with a narrow therapeutic index because in such cases, high inter-individual variability can lead to a substantial fraction of individuals experiencing adverse effects with little or no therapeutic benefit (Tamargo et al., 2015). For environmental chemicals, our results build on previous work in lymphoblastoid cells suggesting that population-based *in vitro* data can fill this crucial gap in chemical safety assessments, extending the approach to organotypic cell models and endpoints with clear relevance to *in vivo* phenotypes. Investigations are underway to apply this approach across a wider set of chemical treatments, including both pharmaceuticals and environmental chemicals. Additional future directions include testing larger populations of iPSC-derived cells as well as incorporating cells from individuals with congenital disorders. Overall, we believe that using population-based iPSCs represents a major leap in the ability to address inter-individual variability in responses to xenobiotics, and therefore has the potential both to substantially decrease the use of animals in research and safety evaluation, increase the net benefits of drugs to patients, as well as to decrease the adverse impacts of environmental chemicals on public health.

## Supplementary Material

Supplemental

## Figures and Tables

**Tab. 1:** Donor Selection and Population Characteristics of iPSC-derived Cardiomyocytes

#	ID	Source	Gender	Ethnicity	Catalogue Number
1	1434	Cellular Dynamics	Female	Caucasian/White: Non-Hispanic	CMC-100–010-001
2	1279	Cellular Dynamics	Male	Caucasian/White: Non-Hispanic	DDP-CMC-1X 01279.107
3	1063	NHLBI	Male	Caucasian/White: Non-Hispanic	DDP-CMC-0.5X 01063.706
4	1064	NHLBI	Female	Caucasian/White: Non-Hispanic	DDP-CMC-0.5X 01064.706
5	1070	NHLBI	Female	Caucasian/White: Non-Hispanic	DDP-CMC-0.5X 01070.700
6	1075	NHLBI	Male	Caucasian/White: Non-Hispanic	DDP-CMC-0.5X 01075.700
7	1082	NHLBI	Female	Black: Non-Hispanic	DDP-CMC-0.5X 01082.726
8	1083	NHLBI	Female	Black: Non-Hispanic	DDP-CMC-0.5X 01083.758
9	1118	NHLBI	Male	Black: Non-Hispanic	DDP-CMC-0.5X 01118.704
10	1134	NHLBI	Female	Black: Non-Hispanic	DDP-CMC-0.5X 01134.701
11	1228	NHLBI	Male	Caucasian/White: Non Hispanic	DDP-CMC-0.5X 01228.716
12	1243	NHLBI	Male	Caucasian/White: Non-Hispanic	DDP-CMC-0.5X 01243.701
13	1258	NHLBI	Female	Caucasian/White: Non-Hispanic	DDP-CMC-0.5X 01258.701
14	1299	NHLBI	Male	Caucasian/White: Non-Hispanic	DDP-CMC-0.5X 01299.704
15	1307	NHLBI	Female	Caucasian/White: Non-Hispanic	DDP-CMC-0.5X 01307.704
16	1308	NHLBI	Female	Caucasian/White: Non-Hispanic	DDP-CMC-0.5X 01308.716
17	1318	NHLBI	Male	Caucasian/White: Non-Hispanic	DDP-CMC-0.5X 01318.700
18	1319	NHLBI	Male	Caucasian/White: Non-Hispanic	DDP-CMC-0.5X 01319.755
19	1348	NHLBI	Male	Caucasian/White: Non-Hispanic	DDP-CMC-0.5X 01348.716
20	1364	NHLBI	Female	Caucasian/White: Non-Hispanic	DDP-CMC-0.5X 01364.701
21	1368	NHLBI	Male	Caucasian/White: Non-Hispanic	DDP-CMC-0.5X 01368.716
22	1392	NHLBI	Male	Caucasian/White: Non-Hispanic	DDP-CMC-0.5X 01392.734
23	1395	NHLBI	Male	Caucasian/White: Non-Hispanic	DDP-CMC-0.5X 01395.701
24	1402	NHLBI	Female	Caucasian/White: Non-Hispanic	DDP-CMC-0.5X 01402.701
25	1405	NHLBI	Female	Caucasian/White: Non-Hispanic	DDP-CMC-0.5X 01405.701
26	1406	NHLBI	Male	Caucasian/White: Non-Hispanic	DDP-CMC-0.5X 01406.701
27	1290	NHLBI	Male	Caucasian/White: Non-Hispanic	DDP-CMC-0.5X 01290.755

## Footnotes

1 doi:10.14573/altex.1805301s

## Abbreviations

%CV: Coefficient of Variation
ATP1A1: ATPase Na+/K+ transporting subunit alpha 1
ATP1B1: ATPase Na+/K+ transporting subunit beta 1
CALM3: calmodulin 3
CAMK2B: Calcium/calmodulin-dependent protein kinase type II beta chain
C_max_: Maximum Concentration in Blood Plasma
DMSO: Dimethyl Sulfoxide; hERG, human Ether-à-go-go-Related Gene
ITPR2: inositol 1,4,5-trisphosphate receptor, type 2
iPSC: Induced Pluripotent Stem Cell
IVIVE: *In Vitro*-to-*In Vivo* Extrapolation
PLN: phospholamban
KCNK1: potassium channel subfamily K member 1
PRKACA: catalytic subunit α of protein kinase A
RED: rapid equilibrium dialysis; RFU, Relative Fluorescence Units
