# Supplementary material for: A human population-based organotypic in vitro model for cardiotoxicity screening
Source: ALTEX. Author manuscript; Available in PMC 2018 Nov 12. (PMC6231908; doi:10.14573/altex.1805301)
Supplement: Supplemental [file NIHMS994287-supplement-Supplemental.pdf]

Grimm et al.:

A human population-based organotypic *in vitro* model for cardiotoxicity screening

Supplementary Data

Tab. S1: Instrumental Parameters for Chemical Analysis by HPLC/MS.

Chemical Name	CAS #	Mode	MRM[a]	Dw[b]	F[c]	CE[d]	CAV[e]
Cisapride	260779-88-2	+	466/184	30	110	30	4
Sotalol	959-24-0	+	273/255	30	110	10	4
			273/213*	30	110	20	4
Propranolol	318-98-9	+	260/183	30	110	20	4
			260/116*	30	110	20	4
Isoproterenol	5984-95-2	+	212.1/194	30	82	9	4
			212.1/152*	30	82	17	4
			212.1/107*	30	82	33	4

[a]MRM = MS/MS ion transitions (amu); [b]Dw = Dwell (msec); [c]F = Fragmentor (Volts); [d]CE = Collision Energy (Volts); and [e]CAV = Cell Accelerator voltage (Volts).
Additional MS parameters are as follows: Ion spray voltages were +3500 V for positive ion analysis; Gas temperature was set to 300^{0}C; Gas flow set to 10l/min; nebulizer set to 35psi; sheath gas temperature set to 350^{0}C with a gas flow of 11l/min; nozzle voltage set to 1000 V. Qualifier parameters for analytes marked with an *.

doi:10.14573/altex.1805301s

Fig. S1: Scatter plot and correlation matrix for baseline cardiophysiological phenotype measurements
Data from Fig. 1 are plotted as scatter plots in the lower left, with the correlation coefficient (absolute value) shown in the upper right. BPM=beats per minute; CV = coefficient of variation.

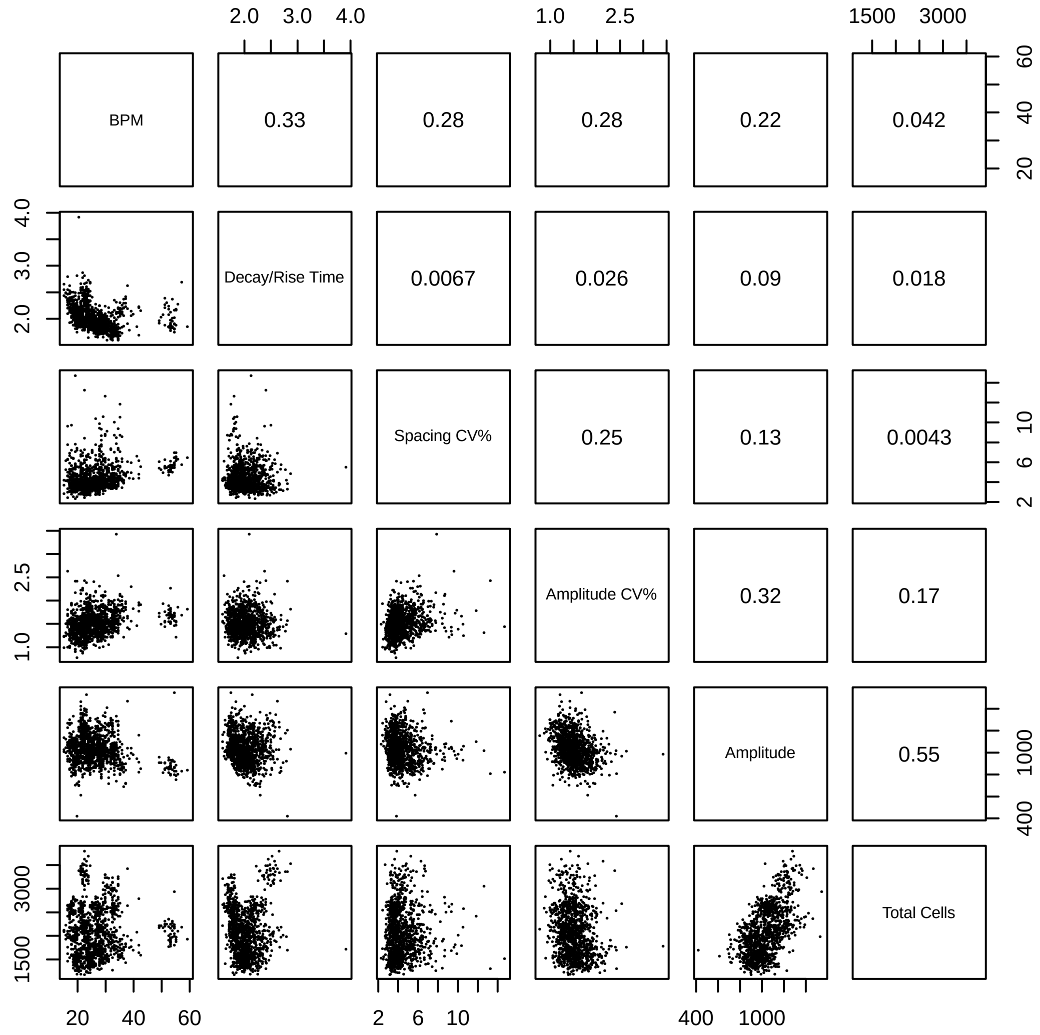

Fig. S2: Drug-induced gene expression differences between donors
Venn diagrams of overlapping differentially expressed gene sets by iPSC cardiomyocyte donor based on data shown in Fig. 7.

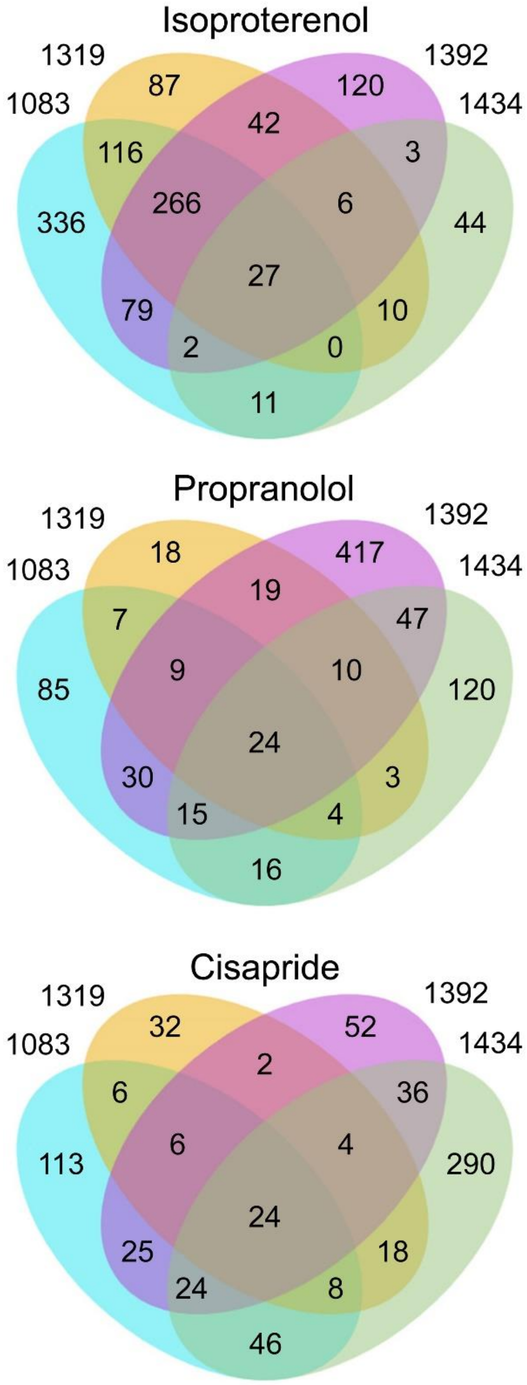

This is an Open Access article distributed under the terms of the Creative Commons Attribution 4.0 International license (http://creativecommons.org/licenses/by/4.0/), which permits unrestricted use, distribution and reproduction in any medium, provided the original work is appropriately cited.
